# BLDC motor’s speed and torque modelling through hybrid machine learning based approach of nonlinear autoregressive neural network with exogenous inputs (NARX-NN)

**DOI:** 10.1371/journal.pone.0333080

**Published:** 2025-11-21

**Authors:** Muhammad Aseer Khan, Husan Ali, Dur-e-Zehra Baig, Fahad R. Albogamy

**Affiliations:** 1 Department of Electrical and Avionics Engineering, Air University Aerospace and Aviation Campus Kamra, Attock, Pakistan; 2 Faculty of Electrical Engineering, Ghulam Ishaq Khan Institute of Engineering Sciences and Technology, Topi, Swabi, Pakistan; 3 Computer Sciences Program, Mathematics Department, Turabah University College, Taif University, Taif, Saudi Arabia; Southwest University of Science and Technology, CHINA

## Abstract

Modeling the complex nonlinear dynamics of Brushless DC motors has been a prominent research focus over the past two decades, driven by their superior advantages and widespread industrial applications. Despite extensive efforts, achieving high-efficiency prediction of speed and torque responses remains a challenge. This study proposes a hybrid machine learning-based approach using the Nonlinear Autoregressive Neural Network with Exogenous Inputs. The method combines artificial neural networks and system identification techniques to enhance predictive accuracy in nonlinear dynamic systems. For both speed and torque modeling, optimal time delays and neural network layer sizes are selected to accurately capture the ripple effects under a multi-step input signal applied to a three-phase inverter. The proposed models yield Mean Square Error values as low as ${\text{1}\text{0}}^{-\text{4}}$ for speed and ${\text{1}\text{0}}^{-\text{3}}$ for torque. Regression coefficients of 1.000 for speed and 0.998 for torque are achieved consistently across training, validation, testing, and additional testing phases, following a data split of 70% for training and 15% each for validation and testing. To further evaluate generalization, the approach is tested using a distinct multi-step input voltage signal, with the results confirming the robustness and superiority of the proposed method in both speed and torque prediction. Comparative analysis with existing literature demonstrates the dominance of the proposed models. These high-fidelity models can serve as a foundation for designing advanced controllers aimed at efficient speed regulation and torque ripple mitigation in Brushless DC motors.

## 1. Introduction

The modern industrial landscape has been significantly shaped by electric motors, particularly DC motors, which remain vital in diverse industrial applications. Among these, brushless DC (BLDC) motors have gained prominence due to their high efficiency, fast dynamic response, and minimal maintenance needs, resulting from the elimination of brushes and mechanical commutators [1–3]. Structurally, BLDC motors feature a permanent magnet rotor and trapezoidal back-EMF waveform, making them suitable for motion-control applications in electric vehicles, robotics, and biomedical systems [4–7]. However, a critical drawback in BLDC motor operation is torque ripple, primarily caused by current ripple, cogging torque, and non-ideal back-EMF waveforms [2,7]. These ripples contribute to vibration, acoustic noise, and mechanical stress, which reduce operational lifespan and reliability [8]. BLDC motors are nonlinear systems requiring a three-phase inverter and electronic commutation. Modeling their dynamics involves accounting for various nonlinearities, including commutation effects [9,10], cogging torque [11], and disturbances. While some conventional linear models [12] attempt simplifications, they often fail to capture the real-world behavior of BLDC motors under dynamic loading. Accurate modeling requires precise parameter identification. Studies have explored techniques such as step voltage response [13], torque sensor-based measurements [14], and least squares algorithms [15–17]. These methods vary in cost and accuracy. Furthermore, modeling speed and torque characteristics is essential for performance optimization [18]. Research has investigated system responses to different configurations [19,20], with modeling strategies ranging from transfer functions [21], state-space models [22], and hybrid estimation methods [23]. In [24], a discrete nonlinear matrix-vector model was proposed, while [25] used real-time experimentation and Arduino-based data acquisition to identify speed behavior. Regulation approaches using PI control and PWM voltage variation are also studied in [26]. Artificial Neural Networks (ANNs) are widely used for modeling BLDC behavior [27,28], but most efforts focus only on speed, neglecting torque ripple. For instance, [27] used NARX and least squares to model speed under various voltage profiles but excluded torque. [28] explored speed and efficiency based on motor geometry. [29–31] focused on state prediction or back-propagation methods, but reported low speed accuracy or lacked dynamic torque handling. Other works [32,33] addressed speed modeling via zero-crossing back-EMF detection, which limits ripple estimation.

Despite this progress, many existing models suffer from critical limitations most rely on single-step inputs, which fail to excite full system dynamics [13,27], few studies attempt to model both speed and torque ripple under multi-step voltage inputs, which are more realistic for operational conditions, physics-based models are limited by oversimplified assumptions [34], prior studies often fail to generalize well under noisy or unseen input profiles [35].

To overcome these limitations, this study proposes a hybrid data-driven modeling approach using the Nonlinear Autoregressive Neural Network with Exogenous Inputs (NARX-NN). Unlike Feedforward ANN (FF-ANN), which captures static mappings, or Recurrent Neural Networks (RNNs), which are resource-intensive and prone to vanishing gradients, NARX-NN explicitly models temporal dependencies by incorporating delayed inputs and outputs [36–38]. This makes it highly effective for modeling nonlinear, time-varying systems such as BLDC motors. While deep learning models like LSTM and GRU have shown promise for sequential data, their computational demands and training data requirements limit real-time applicability in embedded systems [39]. NARX-NN offers a better trade-off between accuracy, complexity, and real-time feasibility. [40] predicted the speed and torque of BLDC motor separately using NARX-NN technique, but in that case, the two parameters of DC input voltage and mechanical input torque are taken as input training features. The hidden layer size of 20 and time delay of 2 is used for both separate cases. But the problem lies here is that only relevant features for each speed and torque should be chosen who have a correlation with each of the output. In this paper, for speed, only phase voltages, phase currents and load torque are chosen as only relevant feasible features while for torque, only electromotive force and phase current are chosen as only relevant feasible features. Due to less input features for torque, definitely there will be half layer sizes required for torque as compared to speed. Similarly, whenever there will be a change in any of these input features, both the relevant features will be clearly effected due to that change.

In this study, we extend the classical NARX-NN by incorporating preprocessing techniques, including correlation between several input features and output; hybrid training strategies to avoid overfitting and enhance convergence, multi-step excitation signal design to simulate realistic motor inputs [41].

The proposed method demonstrates high accuracy in capturing both speed and torque ripples, validated through performance metrics such as Regression (R) and Mean Square Error (MSE) across training, validation, testing, and additional test sets. Comparative performance confirms its superiority over classical and ANN-based models, with robust generalization even under dynamic and noisy conditions. The developed model is suitable for embedded deployment and has potential applications in electric vehicle drives, industrial automation, robotic manipulators, and motor health diagnostics [42,43]. Despite its advantages, the proposed approach has limitations. The model does not yet account for temperature-dependent variations or real-time hardware-in-the-loop (HIL) implementation. These aspects are suggested for future work. The key contributions of this study includes a critical assessment of existing BLDC modeling methods, highlighting their shortcomings in ripple modeling under realistic inputs, justification and implementation of NARX-NN over traditional physics-based and alternative ANN models (e.g., FF-ANN, RNN), a novel combination of preprocessing, multi-step excitation, and hybrid training strategies to improve generalization, and demonstration of capability to simultaneously model speed and torque ripples with high accuracy under dynamic input conditions. The NARX-NN model is designed to effectively learn the dynamic and transient characteristics of a system by using past values of both inputs and outputs. Unlike traditional ARX models that are limited to linear relationships, the NARX-NN incorporates nonlinear mappings through a neural network, allowing it to handle more complex system behaviors. By feeding delayed input and output sequences into the network, it learns how the system evolves over time, especially during rapid changes or disturbances. When implemented in a feedback configuration, where the network uses its own predictions as part of the input for the next time step, it becomes even more capable of simulating long-term behaviors. This structure makes it suitable for capturing not only steady-state responses but also intricate transient response. The architecture of the NARX-NN enables it to effectively capture variations in speed and torque, including dynamic behaviors like overshoot and settling time, which are typically not handled well by a basic feedforward artificial neural network. Despite its suitability for nonlinear dynamic systems, the NARX-NN model does come with limitations. It requires careful selection of input/output lags and hidden layer architecture, which may demand iterative tuning. Additionally, its performance can degrade if the training data lacks sufficient excitation or variability, potentially impacting generalization under unseen or noisy conditions. These aspects are further discussed in the conclusion section.

The categorization of this research paper is as follows. Section I gives a thorough introduction and literature review, section II represents the background theory of mathematical model and method adopted, section III gives detail on simulation & results, section IV discusses the results, while section V concludes the study.

## 2. Methods

### 2.1. Mathematical modeling of BLDC motor

A comprehensive BLDC motor dynamical model is given in [23]. The BLDC motor model is composed of two primary components. The first is the electrical section, responsible for computing the electromagnetic torque and motor current. The second is the mechanical section, where the BLDC motor and the inverter equivalent circuit facilitate the rotor’s revolution. The electrical model captures the dynamics of the phase currents, which are governed by Kirchhoff’s Voltage Law (KVL) for each phase. For simplicity, it is assumed that the stator windings are symmetrical, variations in the stator’s self-inductance with rotor position, as well as the mutual inductance between the stator windings, are negligible, stator resistance $R_{s}$ and self-inductance $L_{s}$ are constant.

The back EMF is modeled as a function of rotor position and speed, and represented using flux linkages Φ′.

Applying KVL to phases a, b, and c gives:


$$\frac{di_{a}}{dt}=\frac{\text{1}}{\text{3}L_{s}}(\text{2}v_{ab}+v_{bc}-\text{3}R_{s}i_{a}+\lambda p\omega_{m}(-\text{2}{\Phi^{\prime }}_{a}+{\Phi^{\prime }}_{b}+{\Phi^{\prime }}_{c}))$$ (1)

Similarly, the equations for other phase currents and are obtained by cyclically permuting the phase variables.


$$\frac{di_{b}}{dt}=\frac{\text{1}}{\text{3}L_{s}}(-v_{ab}+v_{bc}-\text{3}R_{s}i_{b}+\lambda p\omega_{m}(-\text{2}{\Phi^{\prime }}_{b}{{+\Phi }^{\prime }}_{c}+{\Phi^{\prime }}_{a}))$$ (2)

The current continuity condition for a balanced three-phase system without a neutral wire requires:


$$\frac{\text{d}{\text{i}}_{\text{c}}}{\text{dt}}=-(\frac{\text{d}{\text{i}}_{\text{a}}}{\text{dt}}+\frac{\text{d}{\text{i}}_{\text{b}}}{\text{dt}})$$ (3)

The electromagnetic torque is expressed in Eq. (4) as:


$$T_{e}=p\lambda (-\text{2}{\Phi^{\prime }}_{a}i_{a}+{\Phi^{\prime }}_{b}i_{b}+{\Phi^{\prime }}_{c}i_{c})$$ (4)

which is derived from the Lorentz force law and the flux linkage expressions.

The rotor angular velocity and position are defined by Equations (5) as:


$$\frac{\text{d}\omega_{\text{m}}}{\text{dt}}=\frac{\text{1}}{\text{J}}({\text{T}}_{\text{e}}-{\text{T}}_{\text{f}}-\text{F}\omega_{\text{m}}-{\text{T}}_{\text{m}})$$ (5)

Rotor position θ is then updated by integrating angular velocity as given in equation (6):


$$\frac{\text{d}\theta }{\text{dt}}=\omega_{\text{m}}$$ (6)

The electrical and mechanical parameters utilized in the aforementioned equations are listed in Table 1.

**Table 1:** Description of electrical and mechanical parameters.

Symbols	Description	Symbols	Description
$L_{s}$	Stator Inductance	p	No. of Pole Pairs
$R_{s}$	Stator Resistance	$T_{e}$	Electromagnetic Torque
$i_{a},i_{b},i_{c}$	Stator Phase Currents	J	Combined inertia of Rotor and load
${\text{v}}_{ab},{\text{v}}_{bc},{\text{v}}_{ca}$	Phase to Phase Voltages	F	Combined viscous friction of rotor and load
$\Phi_{a},\Phi_{b},\Phi_{c}$	Electromotive Forces per unit value to the amplitude of flux	θ	Angular position of rotor
$\omega_{m}$	Angular Velocity	$T_{f}$	
λ	Amplitude of flux induced by rotor permanent magnets in stator phases	$T_{m}$	Mechanical torque at motor shaft

### 2.2. Methodology adopted

**Fig 1:**
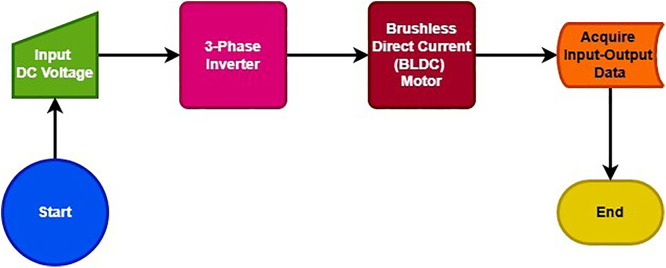
Input-Output Data Acquisition of BLDC Motor.

The BLDC motor comprising of the mathematical equations, presented in the above section, is fed from a 3-Phase inverter, which is given DC input voltage from a DC source. The simulation is carried out for a period of 10 seconds. The basic diagram comprising of how simulation of BLDC motor is carried out in MATLAB/Simulink and data of Speed and Torque is acquired later on using a *simout* block, can be depicted from Fig 1.

**Fig 2:**
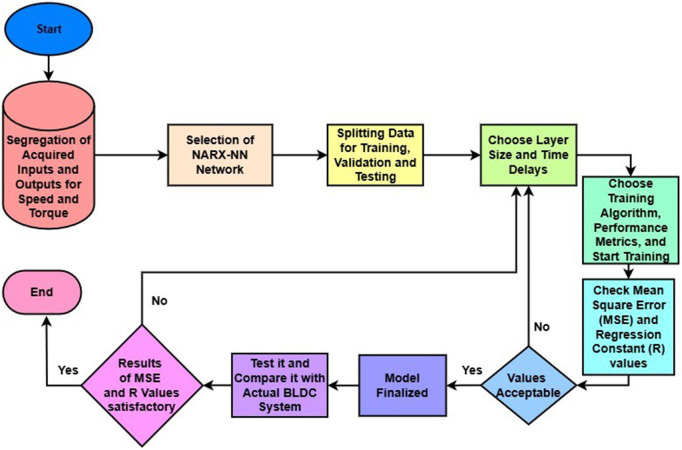
Detailed Methodology in Flowchart.

The detailed methodology after data-acquisition is represented in Fig 2. The acquired dataset is then filtered using only suitable input features. These suitable features are added after finding the correlation using basic correlation function in MATLAB between each feasible input feature and each output speed or torque. It includes phase voltages, phase currents, and Load Torque as input for Speed and Electromotive Force & Phase current as inputs for torque. For modeling purposes, the data is segregated into two sets, one for each speed estimation and torque estimation. The structure of NARX-NN Network is chosen for estimation of each output, is selected due to its ability to handle dynamic time-series data. The data is splitted in to a ratio of 70% for Training and 15% for each validation and Testing.

The hidden layer size of 20 for speed and 10 for torque while 2 time delays for each input and output are chosen heuristically. In this study, the selection of model hyperparameters—such as the number of hidden neurons and time delays—was conducted empirically, guided by domain-specific knowledge of BLDC motor dynamics. This approach is common in engineering-focused applications where resource constraints, deployment feasibility, and model interpretability take precedence over exhaustive optimization. Specifically, as 20 hidden neurons are used for the speed model and 10 for the torque model, along with two-time delays for inputs and outputs. These values were identified through repeated trial-and-error experiments, balancing model complexity and prediction accuracy. While this may not ensure globally optimal configurations, the empirical results demonstrated reliable performance within the tested operational envelope. It is acknowledged that heuristic selection may limit adaptability to significantly different motor configurations or operating environments. However, in motor modeling applications, models are often tuned per system and re-calibrated with minimal data. Moreover, systematic optimization methods (e.g., grid search or Bayesian optimization) are computationally intensive and may not offer significant performance gains relative to the increased cost in practical use cases. Future extensions of this work could incorporate automated hyperparameter tuning strategies to enhance the model’s scalability across diverse motor types and environments, particularly in large-scale or cloud-based predictive maintenance systems.

After that, training algorithm of Levenberg-Marquardt (LM) is chosen due to the reason that it is well suited for optimizing complex nonlinear systems. It does so by combining Gradient Descent and Gauss-Newton methods, which makes it more robust in handling nonlinearities. Furthermore, LM provides fast convergence as compared to standard back propagation algorithms, and it adjusts the time size dynamically, making the training process more stable and reducing the risk of slow convergence. Other advantages include higher accuracy in parameter estimation, avoiding getting trapped in local minima and well suited for Neural Networks.

After selecting these, the training gets started, whenever any of the criteria is met, the training gets stopped. After the training is stopped, MSE and R values are checked, if MSE values are up to ${\text{1}\text{0}}^{-\text{2}}$ and regression values closer to 1 in each training, testing and validation, the values are accepted. If criteria is unmet, the network architecture is re-tuned (adjust layer size, delays) and training is re-initiated. Once performance is satisfactory, the model is finalized and tested with unseen data. If the new test results meet the same performance criteria, the model is considered validated. A high-level overview of the process is illustrated in Fig 2.

### 2.3. Simulation and results

#### 2.3.1. Input-output dataset generation.

**Fig 3:**
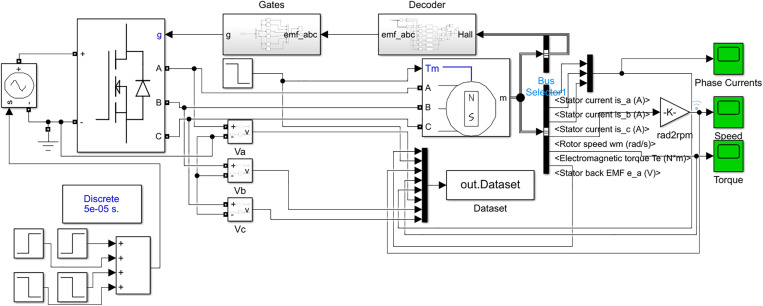
Detailed Simulation of BLDC Motor for Data Acquisition in Simulink.

The Input-Output dataset is generated by using a Discrete Power GUI block of sample time 50e-06 s in simulation. The default values set in the Permanent Magnet Synchronous Machine (PMSM are set. The multi-step input signal is connected to a Controlled Voltage Source (CVS) block, which is then connected to Universal Bridge, which acts as a 3-phase inverter. The default values of block are used, output of block are three phase voltages $V_{a}$, $V_{b}$, and $V_{c}$ given gate signals, phase voltages are then connected BLDC motor block. The output of BLDC motor block are different parameters including stator currents, stator back-emf signals, rotor angular velocity and position, electromagnetic torque with input of load torque and phase voltages. The detailed simulation of BLDC motor in Simulink is shown in Fig 3.

**Fig 4:**
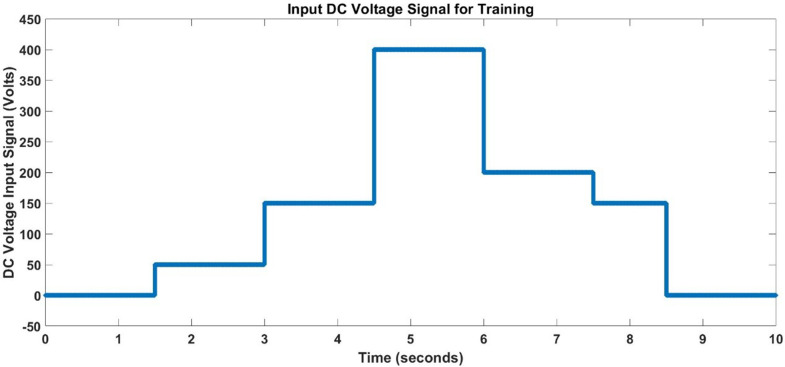
DC Input Voltage for Training.

**Fig 5:**
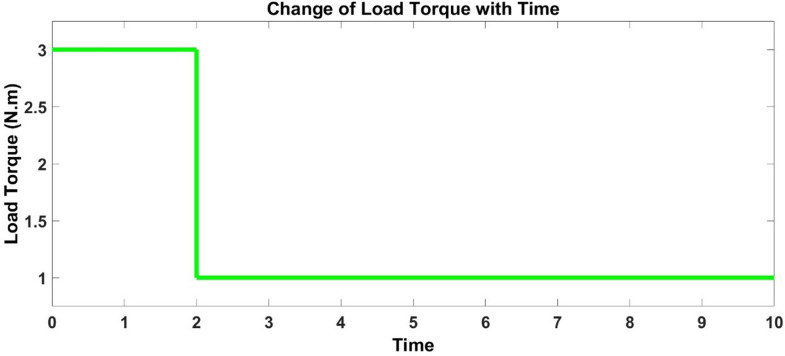
Input Load Torque.

The multi-input DC voltage signal fed to 3-phase inverter during which input and output data is acquired is given below in Fig 4. Similarly, load torque fed to BLDC motor is shown below in Fig 5.

**Fig 6:**
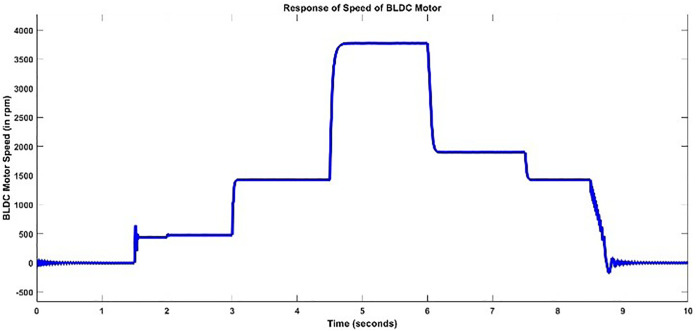
Actual Speed Response for Training.

**Fig 7:**
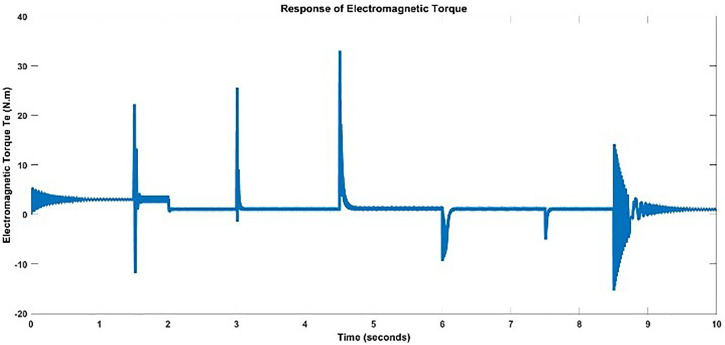
Actual Torque Response for Training.

Speed and torque are two outputs that are to be modeled. The actual speed and torque responses of BLDC motor acquired by providing the above DC voltage signal is shown below in Figs 6 and 7. The ripples in curves can be clearly seen.

A total of 200003 samples for all the inputs and outputs are exported to workspace after carrying out simulation. For each Speed and Torque, the relevant features, i.e., inputs are gathered and a fitness value is acquired of each feature for speed and torque. For Speed modeling, Phase voltages, Phase currents, and Load Torque are taken as training inputs while only Electromotive Force and Phase current are taken training inputs for modeling of Torque. Only the best features with fitness value greater than zero based upon the correlation between the input and output are finalized and given in Table 2, which will be used for training purposes.

**Table 2:** Best features for speed and torque.

Speed	Torque
$V_{a}$	$I_{a}$
$V_{b}$	
$V_{c}$	
$I_{a}$	
$I_{b}$	$E_{a}$
$I_{c}$	
$T_{L}$	

The algorithm, data division, parametric measure, layer size and time delay used for each speed and torque are given in Table 3.

**Table 3:** Tuning parameters for speed and torque model.

Tuning Parameters	Speed	Torque
**Data Division**	Random	Random
**Algorithm Used**	Levenberg-Marquardt (LM)	Levenberg-Marquardt (LM)
**Performance**	Mean Square Error (MSE)	Mean Square Error (MSE)
**Layers**	2	2
**Time Delay**	2	2
**Layer Sizes**	20	10

**Fig 8:**
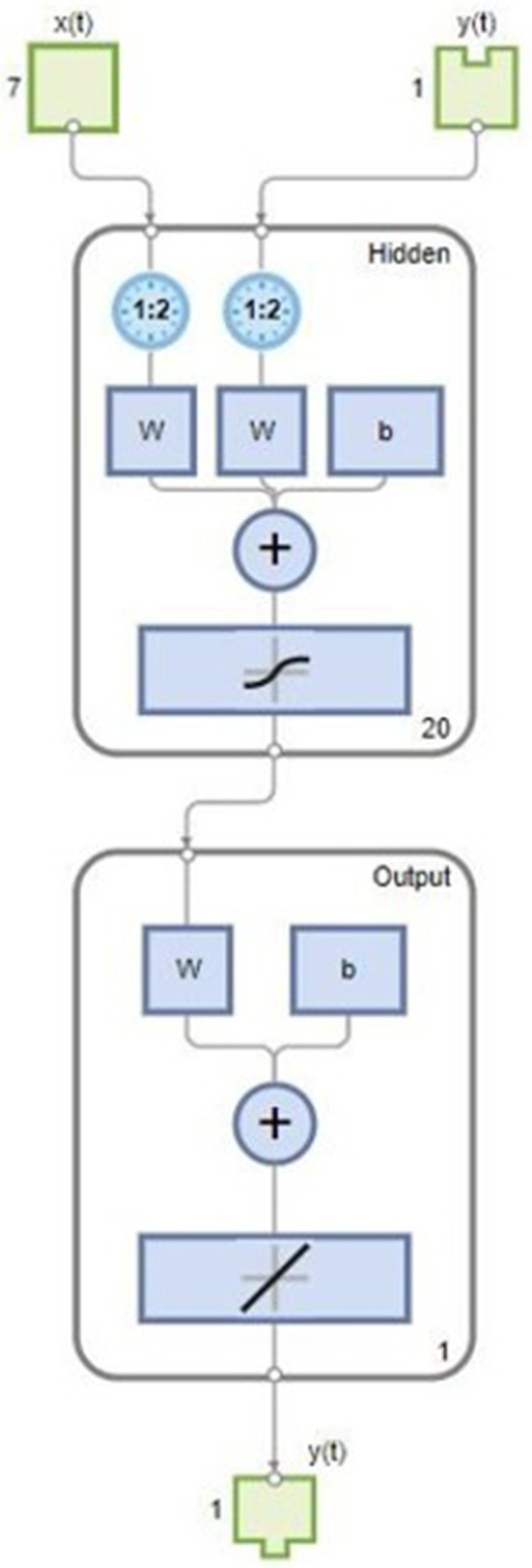
Proposed NARX-NN Structure for Estimation of Speed.

**Fig 9:**
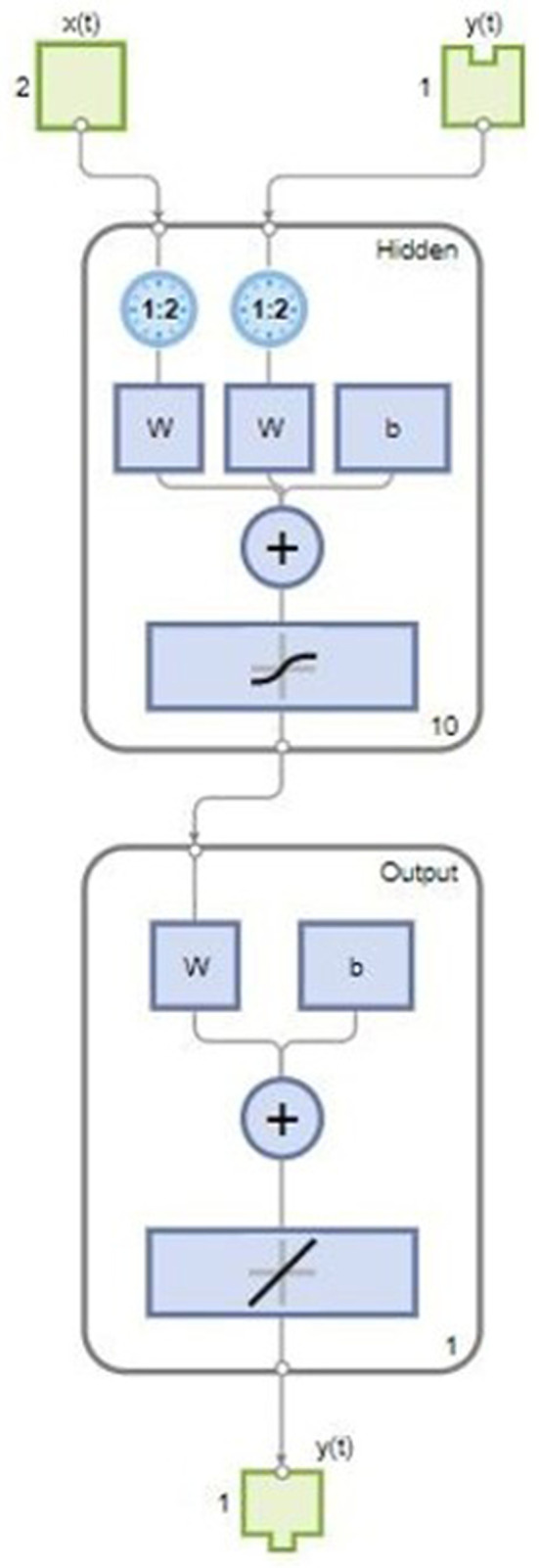
Proposed NARX-NN Structure for Estimation of Torque.

The finalized NARX-NN structure for each speed and torque are represented below in Figs 8 and 9 respectively. For estimation of Speed, the structure given below depicts 7 features, one output response, hidden layer size of 20. It is taking both x(t) and y(t) as inputs because it is taking past values with past time delays of 2 for both inputs and output.

For estimation of Torque, the structure given below depicts 2 features, one output response and hidden layer size of 10.

The training progress results for each speed and torque are shown in Tables 4 and 5 respectively.

**Table 4:** Training progress results for speed.

Unit	Initial Value	Stopped Value	Target Value
Epochs	0	1000	1000
Elapsed Time	–	00:13:30	
Performance	4.06e + 06	0.000305	0
Gradient	2.06e + 07	18.9	1e-07
Mu	0.001	0.01	1e + 10
Validation Checks	0	0	6

**Table 5:** Training progress results for torque.

Unit	Initial Value	Stopped Value	Target Value
Epochs	0	1000	1000
Elapsed Time	–	00:11:29	
Performance	1.52e + 03	0.000899	0
Gradient	4.53e + 03	0.0149	1e-07
Mu	0.001	0.0001	1e + 10
Validation Checks	0	0	6

#### 2.3.2. Modelling results of speed.

**Fig 10:**
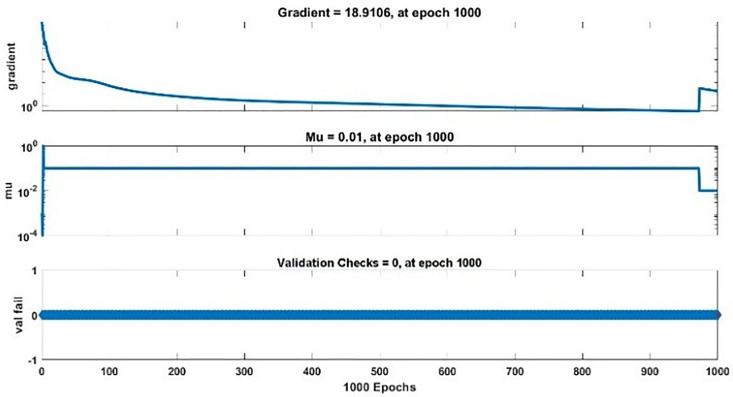
Training State Results of Speed Model.

**Fig 11:**
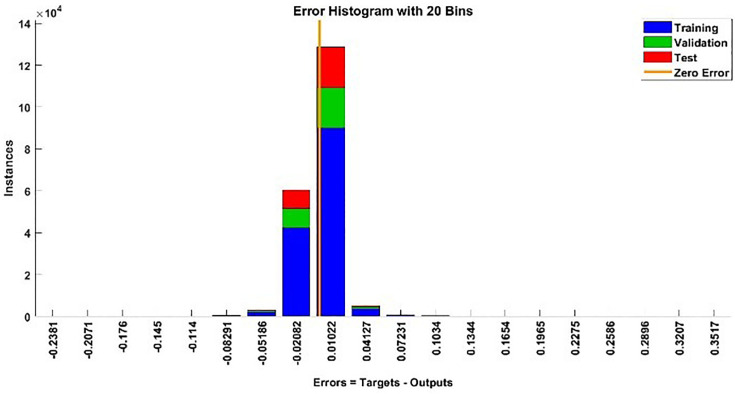
Error Histogram of Speed Model.

**Fig 12:**
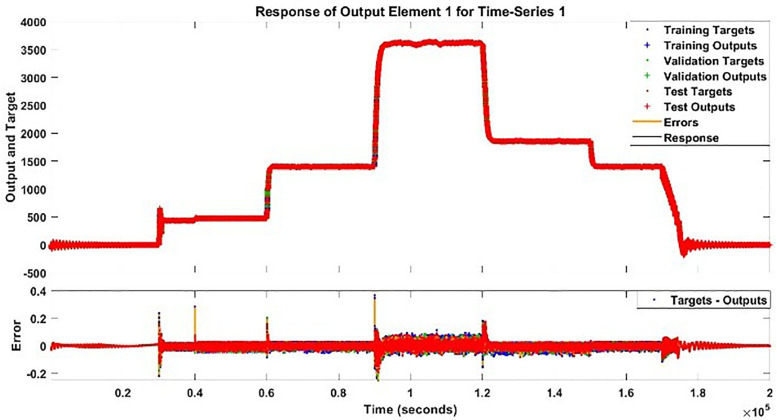
Response Plot of Speed Model.

**Fig 13:**
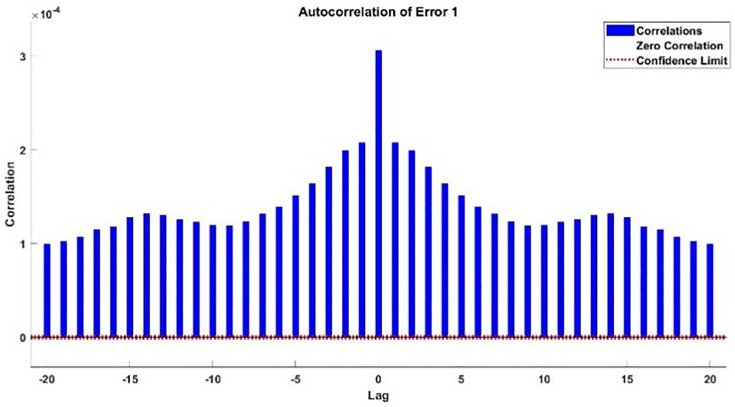
Autocorrelation Error of Speed Model.

The modeling results of Speed model including training state results, error histogram, response plot and Autocorrelation of error are shown below in the Figs 10–13.

#### 2.3.3. Modelling results of torque.

**Fig 14:**
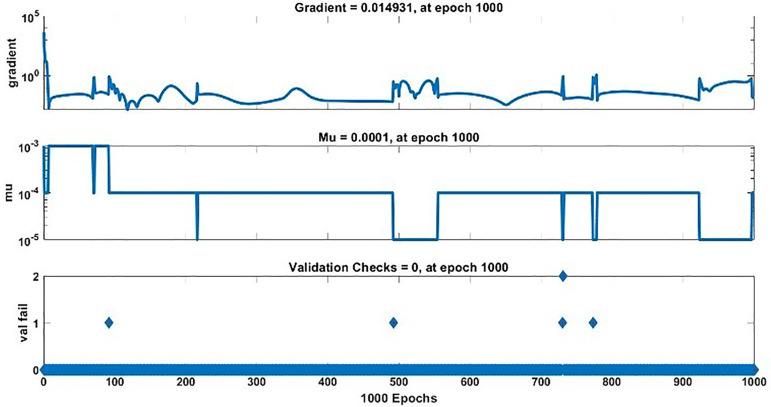
Training State Results of Torque Model.

**Fig 15:**
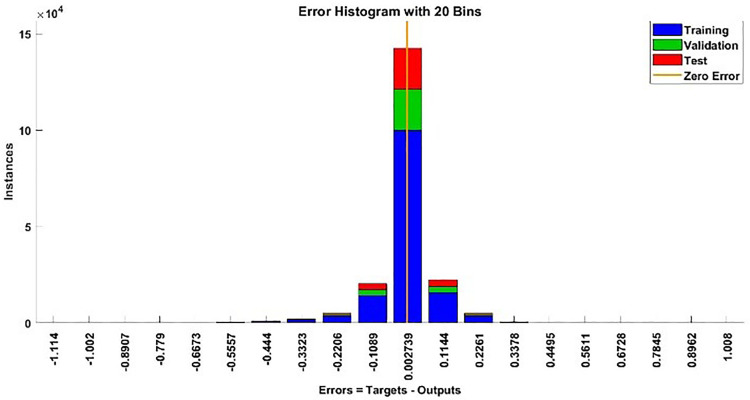
Error Histogram of Torque Model.

**Fig 16:**
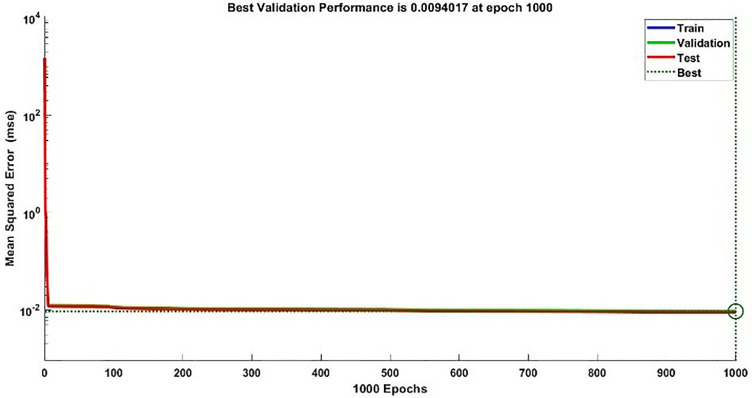
Validation Performance Plot of Torque Model.

**Fig 17:**
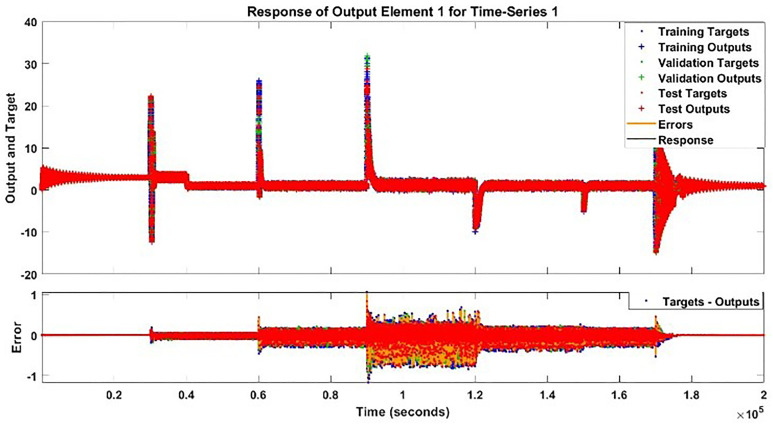
Response Plot of Torque Model.

**Fig 18:**
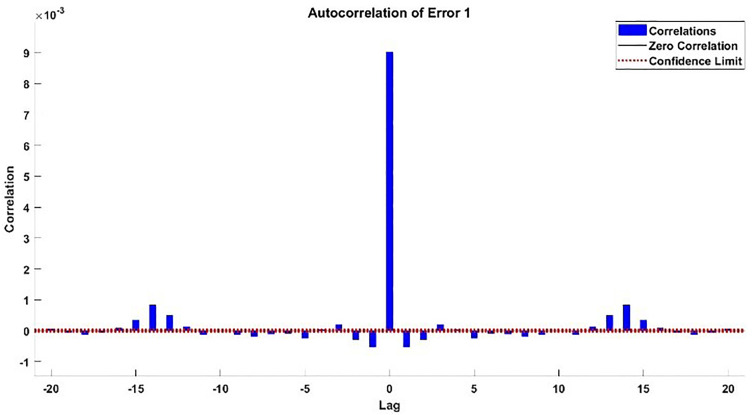
Autocorrelation Error Plot of Torque Model.

The modeling results of Torque model including training state results, error histogram, performance plot, response plot and Autocorrelation of error are shown below in the Figs 14–18.

#### 2.3.4. Additional testing results.

**Fig 19:**
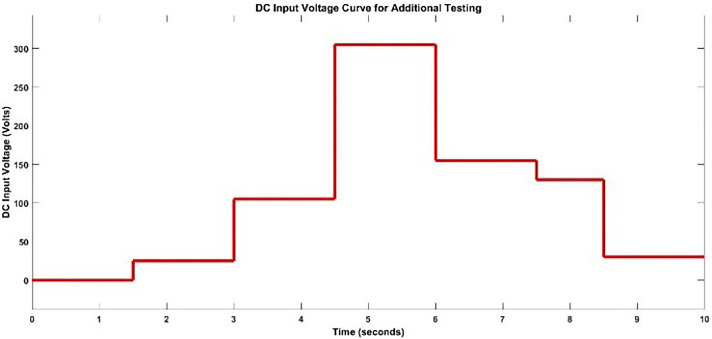
DC Input Voltage Signal for Additional Testing.

For additional testing of both speed and torque models, a unique testing signal is applied with unique steps in the DC input voltage signal to further test the models. The unique testing signal is shown below in the Fig 19.

1) **SPEED TESTING RESULTS**

**Fig 20:**
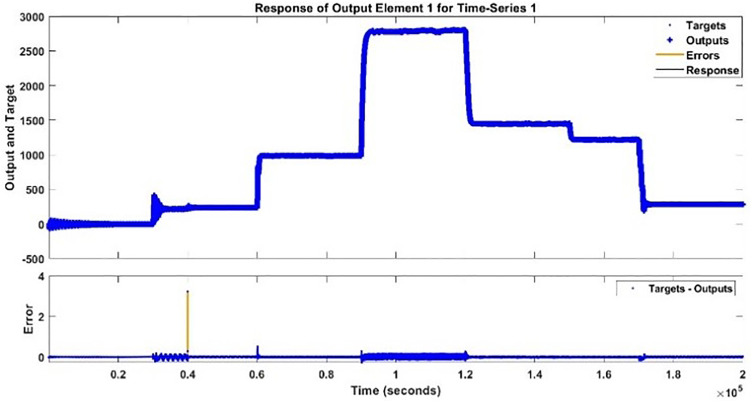
Additional Testing of Speed Model and Actual Speed Response.

The testing signal is applied to both the speed model and the BLDC motor system, a comparison plot is shown between the actual response and the modeled response is Fig 20.

**Fig 21:**
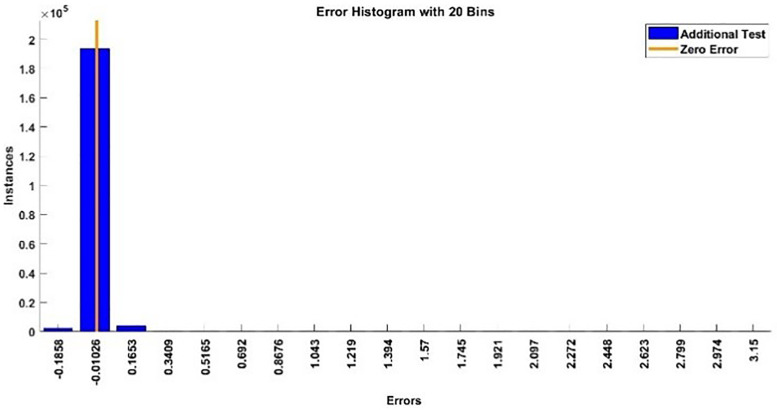
Error Histogram of Additional Testing for Speed.

**Fig 22:**
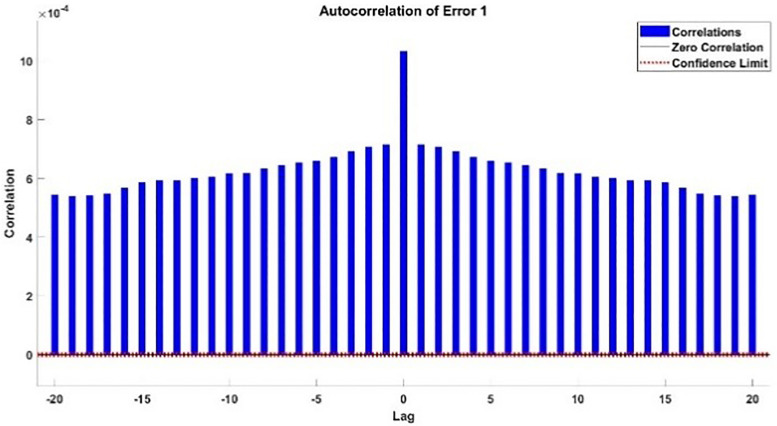
Autocorrelation of Error of Additional Testing.

The error histogram and autocorrelation of error are shown in Figs 21 and 22 respectively.

2) **TORQUE TESTING RESULTS**

**Fig 23:**
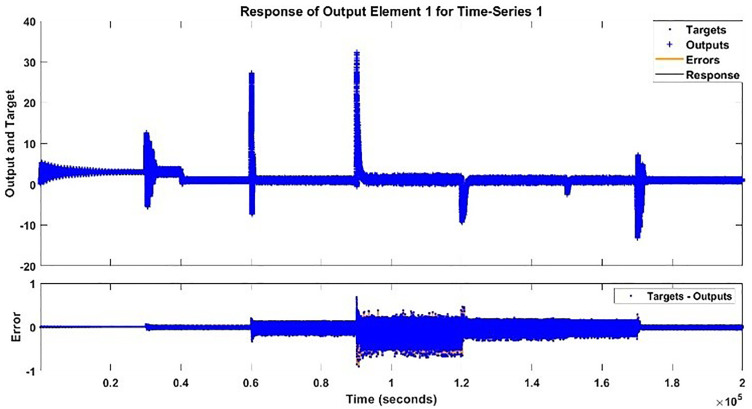
Additional Testing of Torque Model and Actual Torque Response.

**Fig 24:**
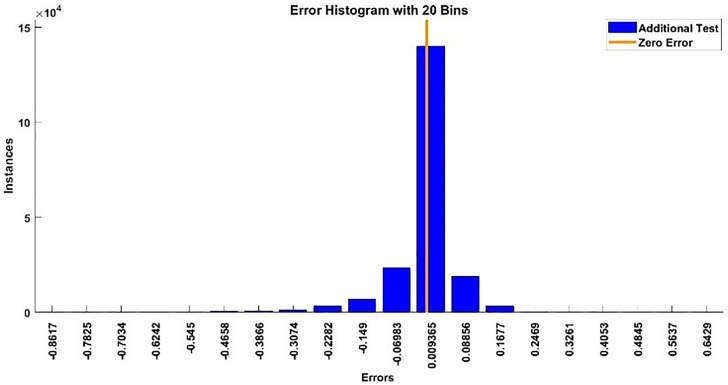
Error Histogram for Additional Testing.

**Fig 25:**
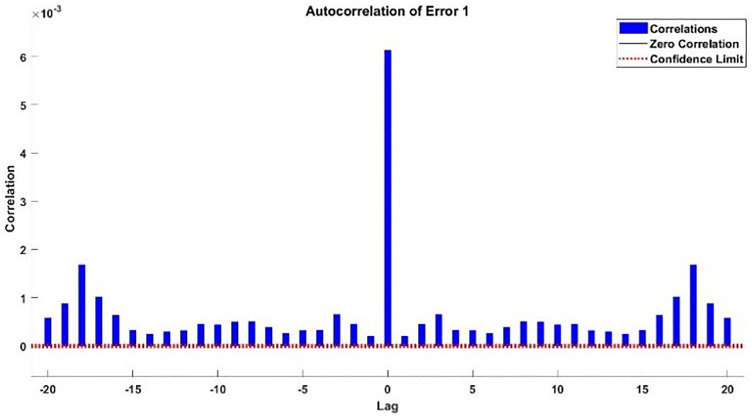
Autocorrelation of Error for Additional Testing.

The testing signal is applied to both the torque model and the BLDC motor system, a comparison plot is shown between the actual response and the modeled response is Fig 23. The error histogram and autocorrelation of error are shown in Figs 24 and 25 respectively.

#### 2.3.5. Combined statistical results.

The acquired combined statistical results for training, validation, testing and additional testing for each estimated model of speed and torque model are presented in Table 6.

**Table 6:** Finalized results of speed and torque models.

	Observations		MSE		R	
	S	T	S	T	S	T
Training	140001		3.0469e-04	0.0090	1	0.9988
Validation	30000		3.1264e-04	0.0094	1	0.9986
Testing	30000		3.0949e-04	0.0089	1	0.9988
Additional Testing	20003		3.0916e-04	0.0061	1	0.9988

#### 2.3.6. Discussion and analysis.

One can analyze from the plots of Speed and Torque that whenever DC input voltage changed at a specific instant changed or Load torque is varied, the speed and torque is changed. The Speed and Torque exhibits nonlinear phenomena in BLDC motor, which are dependent on several parameters. To fully capture the dynamics of BLDC motor, is a trivial process.

The proposed hybrid ML based modelling approach using NARX-NN captured the dynamics of both speed and torque of BLDC motor very efficiently including transient and steady state behavior along with capturing all the ripples too. If we take in to account all the factors for both modelling, all the results whether graphic or statistically, NARX-NN emerges to be powerful tool for estimating the Speed and Torque of BLDC motor. Both models depicted superb performance in terms of MSE and R values in each training, testing, validation and additional testing. Speed model has R values of ‘1’ while Torque Estimator has R values equal to 0.9988, similarly, for speed model, MSE values acquired are up to ${\text{10}}^{-\text{4}}$ for Speed model and ${\text{10}}^{-\text{3}}$ for Torque model. For additional testing, MSE values up to ${\text{10}}^{-\text{4}}$for Speed and 0.0061 value for Torque is obtained while ‘R’ value of 1 is obtained in case of speed model and R value of 0.9988 is obtained for Torque model. The values obtained directly reflects the exact match between the modelled response and actual response of BLDC motor for each speed and torque. The values thus obtained in this research study are far better than statistical results obtained in [29,30], in which only 83.34% accuracy is achieved using a third-order transfer function model and 90% accuracy respectively for speed prediction. Similarly, the results are far better than [27] in which 98% validation accuracy is achieved for prediction of speed of BLDC motor. A comparison table comparing results with existing techniques and methods in the literature review is given in Table 7.

**Table 7:** Comparison table.

Method/Technique Applied for Modelling	Output	MSE	R-Value	Prediction Accuracy
ANN [28]	Speed	0.001849	–	–
TF Based Modelling [29]	Speed	–	–	83.34%
Grey Box Model Estimation [30]	Speed	–	–	90%
ANN [31]	Speed	1.5	0.9801	98%
BEMF ZCD: Typical [32,33]	Speed	–	0.998	99.84%
Proposed NARX-NN Structure	Speed	0.00030949	1	99.999%
	Torque	0.0061	0.9988	99.98%

From the above modelling statistical results, shown in the table, it can be seen that the proposed NARX-NN framework accurately estimated the BLDC motor speed and torque with very low MSE and high R values across all datasets. Further testing it on additional test data further confirms the model robustness as it obtained the same MSE and R values. The error autocorrelation and histograms demonstrate the statistical soundness of residuals. When compared with traditional and AI-based techniques from literature, the proposed approach outperforms its superiority in terms of both accuracy and reliability. There is no any work done on the prediction of Torque of BLDC motor in the literature whose metrics can be compared.

The proposed NARX-NN can be used to design controllers with ease. In future, in the first option, Model Predictive Control (MPC) based on proposed NARX-NN model can be designed. The NARX-NN model can be used to predict future speed and torque responses based on current and past inputs. The control inputs (PWM duty cycle, voltage) can be optimized to minimize the speed and torque errors. Then a cost function can be implemented to ensure the smooth control actions and minimization of torque ripples. In the second option, Adaptive Proportional-Integral (PI)/Sliding Mode Controller can be tuned. The proposed NARX-NN model will helps in real-time estimation of system parameters, allowing the dynamic tuning of a PI or Sliding Mode Controller (SMC). Adaptive gain adjustment to get improved performance under load torque variations and disturbances can be achieved. For a third option, Reinforcement Learning (RL)-based optimization can be used. RL or Deep Learning (DL) techniques can be used to refine control actions in it. The RL agent will interact with the proposed NARX-NN model to learn optimal control strategies which will balance speed regulation and torque ripple minimization.

## 5. Conclusion

This study presents a hybrid machine learning approach based on the NARX neural network to effectively model the nonlinear dynamics of a Brushless DC (BLDC) motor, capturing both transient and steady-state behavior with high accuracy. The proposed model employs a two-layer neural network architecture, utilizing a hidden layer with 20 neurons and two time delays for both input and output, integrating the ARX model structure within a neural network framework. The dataset was divided into 70% for training and 15% each for validation and testing. The developed speed and torque models demonstrated excellent predictive performance, achieving Mean Square Error (MSE) values as low as ${\text{1}\text{0}}^{-\text{4}}$ for speed and ${\text{1}\text{0}}^{-\text{3}}$ for torque. Regression (R) values reached 1.000 for the speed model and 0.998 for the torque model across all data partitions, including additional testing, confirming the robustness and reliability of the proposed method. Further validation was conducted using a novel multi-step input signal, with the results reaffirming the effectiveness of the approach. When compared with existing studies on BLDC motor modeling, the proposed technique outperforms previous methods in terms of accuracy and generalization, making it a strong candidate for future applications in control system design for speed regulation and torque ripple mitigation.

## Supporting information

S1 File Dataset_of_Variables. (MAT)

## Abbreviations

BLDC: Brushless DC Motor
ML: Machine Learning
NARX-NN: Modelling, Nonlinear Autoregressive Neural Network with Exogenous Inputs
SI: System Identification

## References

[1] Hameed HS. “Brushless DC Motor Controller Design Using Matlab Applications,” 3rd Scientific Conference of Engineering Science (ISCES). 2018.

[2] Senthilnathan A, Palanivel P, Kumar KR. Mathematical Modelling and Torque Ripple Waning in BLDC Motor Using Outgoing-Phase Current Discharge Hysteresis Controlled ANFIS Controller. Mathematical Problems in Engineering. 2022;2022:1–21. doi: 10.1155/2022/3971695

[3] Pandey MK, Tripathi A, Dwivedi B. A technique to minimize the effect of current harmonics in a brushless DC motor drive, Proceedings of the IEEE 10th Conference on Industrial Electronics and Applications. Auckland, New Zealand: IEEE; 2015. p. 702–6. doi: 10.1109/iciea.2015.7334199

[4] Peixo ZMA, Freitas SFM, Seixas PF, Menezes BR, Cortizo PC, Lacerda WS. Application of sliding mode observer for induced E.M.F., position and speed estimation of permanent magnet motors, 2, Proceedings of the international conference on power electronics and drive systems. Singapore: IEEE; 1995. p. 599–604.

[5] Lee JG, Park CS, Lee JJ, Lee GH, Cho HO, Hong JP. Characteristic analysis of brushless motor considering drive type. In: Proceedings of the KIEE Summer Annual Conference. Jeju, Republic of Korea. 2002. p. 589–91.

[6] Carlson R, Lajoie-Mazenc M, Fagundes JC d. S. Analysis of torque ripple due to phase commutation in brushless DC machines. IEEE Trans on Ind Applicat. 1992;28(3):632–8. doi: 10.1109/28.137450

[7] Li W, Fang J, Li H, Tang J. Position Sensorless Control Without Phase Shifter for High-Speed BLDC Motors With Low Inductance and Nonideal Back EMF. IEEE Trans Power Electron. 2016;31(2):1354–66. doi: 10.1109/tpel.2015.2413593

[8] Lin Y-K, Lai Y-S. Pulsewidth Modulation Technique for BLDCM Drives to Reduce Commutation Torque Ripple Without Calculation of Commutation Time. IEEE Trans on Ind Applicat. 2011;47(4):1786–93. doi: 10.1109/tia.2011.2155612

[9] LEE B-K, EHSANI M. Advanced Simulation Model for Brushless DC Motor Drives. Electric Power Components and Systems. 2003;31(9):841–68. doi: 10.1080/15325000390227191

[10] Kim I, Nakazawa N, Kim S, Park C, Yu C. Compensation of torque ripple in high performance BLDC motor drives. Control Engineering Practice. 2010;18(10):1166–72. doi: 10.1016/j.conengprac.2010.06.003

[11] Lu H, Zhang L, Qu W. A New Torque Control Method for Torque Ripple Minimization of BLDC Motors With Un-Ideal Back EMF. IEEE Trans Power Electron. 2008;23(2):950–8. doi: 10.1109/tpel.2007.915667

[12] Shanmugasundram R, Zakaraiah KM, Yadaiah N. Modeling, simulation and analysis of controllers for brushless direct current motor drives. Journal of Vibration and Control. 2012;19(8):1250–64. doi: 10.1177/1077546312445200

[13] Wu W. DC Motor Parameter Identification Using Speed Step Responses. Modelling and Simulation in Engineering. 2012;2012:1–5. doi: 10.1155/2012/189757

[14] Choi J-H, You S-H, Hur J, Sung H-G. The design and fabrication of BLDC Motor and Drive for 42V automotive applications, Proceedings of the IEEE International Symposium on Industrial Electronics (ISIE ′07). 2007. p. 1086–91. doi: 10.1109/isie.2007.4374749

[15] Kapun A, Čurkovič M, Hace A, Jezernik K. Identifying dynamic model parameters of a BLDC motor. Simulation Modelling Practice and Theory. 2008;16(9):1254–65. doi: 10.1016/j.simpat.2008.06.003

[16] Blauch AJ, Bodson M, Chiasson J. High-speed parameter estimation of stepper motors. IEEE Trans Contr Syst Technol. 1993;1(4):270–9. doi: 10.1109/87.260272

[17] Patankar R, Zhu L. Real-time multiple parameter estimation for voltage controlled brushless DC motor actuators, 4, Proceedings of the American Control Conference (AAC ′04). 2004. 3851–6.

[18] “Introduction to Torque and Speed Characteristics of BLDC Motor,” Lunyee.com. 2024. [accessed Mar. 16, 2025]. https://www.lunyee.com/news/faq/introduction-to-torque-and-speed-characteristics-of-bldc-motor.html

[19] Manda P, Veeramalla SK. Brushless DC Motor Modeling and Simulation in the MATLAB/SIMULINK Software Environment. AMA_B. 2021;64(1–4):27–33. doi: 10.18280/ama_b.641-404

[20] Samitha Ransara HK, Madawala UK. Modelling and analysis of a low cost Brushless DC motor drive,” 2013 IEEE International Conference on Industrial Technology (ICIT), Cape Town, South Africa. 2013. p. 356–61. doi: 10.1109/icit.2013.6505698

[21] Navidi N, Bavafa M, Hesami S. A new approach for designing of PID controller for a linear brushless DC motor with using ant colony search algorithm. IEEE Power & Energy Engineering c onference. 2009. 1–5.

[22] Muratti V, Kulkarni V, Bhat S, Rajur S, Laddigatti A. Mathematical Modelling and Simulation of BLDC Motor with Trapezoidal Control Technique,” 2023 International Conference on Smart Systems for applications in Electrical Sciences (ICSSES). 2023. doi: 10.1109/icsses58299.2023.10199918

[23] Xiang C, Wang X, Ma Y, Xu B. Practical Modeling and Comprehensive System Identification of a BLDC Motor. Mathematical Problems in Engineering. 2015;2015:1–11. doi: 10.1155/2015/879581

[24] Trefilov S. Non-linear discrete model of BLDC motor for studying the range of permissible values of the voltage vector in the state space. MATEC Web Conf. 2020;329:03070. doi: 10.1051/matecconf/202032903070

[25] Md Zain BA, Anuar F, Latif I. Modeling and speed control for sensorless DC motor BLDC based on real time experiment. 2019.

[26] Porselvi T, Aouthithiye Barathwaj SY, CS SG, Tresa Sangeetha SV, Shalini Priya J. Deep Learning Based Predictive Analysis of BLDC Motor Control,” 2022 IEEE 3rd Global Conference for Advancement in Technology (GCAT). Bangalore, India. 2022. p. 1–6. doi: 10.1109/gcat55367.2022.9972193

[27] Khan MA, Baig DZ, Ali H. et al. Optimized System Identification (SI) of Brushless DC (BLDC) motor using Data-Driven Modeling Methods. Sci Rep 15. 2025;8497. doi: 10.1038/s41598-025-93444-0 PMC11904227 40074813

[28] Rahmani O, Iltarabian M, Sadrossadat SA. Modeling and Simulation of Speed and Efficiency of BLDC Motor as A Starter Motor Based on Multilayer Perceptron (MLP) Neural Network,” 2018 IEEE Transportation Electrification Conference and Expo, Asia-Pacific (ITEC Asia-Pacific). Bangkok, Thailand. 2018. p. 1–5. doi: 10.1109/itec-ap.2018.8433299

[29] Li H, Wu T, Huang Y. Robust adaptive control for BLDC motors based on parameter identification. Int J Electr Power Energy Syst. 2012;43(1):406–13.

[30] Dasanayake N, Perera S. Motor state prediction and friction compensation for brushless DC motor drives using data-driven techniques. arXiv. 2023. doi: 10.48550/arxiv.2311.16533

[31] Nizam M, Mujianto A, Triwaloyo H, Inayati. Modelling on BLDC motor performance using artificial neural network (ANN),” 2013 Joint International Conference on Rural Information & Communication Technology and Electric-Vehicle Technology (rICT & ICeV-T). Bandung, Indonesia. 2013. p. 1–4. doi: 10.1109/rict-icevt.2013.6741520

[32] Tsotoulidis S, Safacas A. A sensorless commutation technique of a brushless DC motor drive system using two terminal voltages in respect to a virtual neutral potential. In: 20th Int. Conf. Electr. Mach. ICEM. Institute of Electrical and Electronics Engineers Inc.; 2012. p. 830–6.

[33] Togneri M, Rahman M. System identification of a brushless DC motor using multi-step input signals. J Electr Eng Autom. 2012;14(2):93–8.

[34] Rodríguez JBGJA. Identification of the dynamic model of a BLDC motor. Int J Electr Power Energy Syst. 2012;42(1):48–56.

[35] Damodharan P, Vasudevan K. Sensorless Brushless DC Motor Drive Based on the Zero-Crossing Detection of Back Electromotive Force (EMF) From the Line Voltage Difference. IEEE Trans Energy Convers. 2010;25(3):661–8. doi: 10.1109/tec.2010.2041781

[36] Weigend AS, Gershenfeld NA. Time Series Prediction: Forecasting the Future and Understanding the Past. Addison-Wesley; 1994.

[37] Chen S, Billings SA, Luo W. Orthogonal least squares methods and their application to non-linear system identification. International Journal of Control. 1989;50(5):1873–96. doi: 10.1080/00207178908953472

[38] Frank PM, Ding X. Survey of robust residual generation and evaluation methods in observer-based fault detection systems. Journal of Process Control. 1997;7(6):403–24. doi: 10.1016/s0959-1524(97)00016-4

[39] Zhang X, Liu S, Hou Z. Practical challenges in applying deep learning models to embedded motor control systems. IEEE Trans on Industrial Informatics. 2021;17(6):4106–15.

[40] Khan MA, Baig D-E-Z, Ali H, Albogamy FR. Torque and speed prediction of a brushless direct current motor using nonlinear autoregressive with exogenous inputs and neural network. Sci Rep. 2025;15(1):27191. doi: 10.1038/s41598-025-11296-0 40715181 PMC12297312

[41] Asif MT, Ali A. Impact of input signal design on neural network-based system identification of BLDC motors. Journal of Dynamic Systems, Measurement, and Control. 2023;145(4).

[42] Chen H, et al. Application of neural network models in fault diagnosis and control of electric drives: A review. IEEE Transactions on Industrial Electronics. 2021;68(10):9847–58.

[43] Zhan C, et al. Hybrid modeling for electric vehicle motor systems using NARX and particle swarm optimization. Engineering Applications of Artificial Intelligence. 2022;116.

